# Antibodies to Human Herpesviruses in Myalgic Encephalomyelitis/Chronic Fatigue Syndrome Patients

**DOI:** 10.3389/fimmu.2019.01946

**Published:** 2019-08-14

**Authors:** Jonas Blomberg, Muhammad Rizwan, Agnes Böhlin-Wiener, Amal Elfaitouri, Per Julin, Olof Zachrisson, Anders Rosén, Carl-Gerhard Gottfries

**Affiliations:** ^1^Section of Clinical Microbiology, Department of Medical Sciences, Uppsala University, Uppsala, Sweden; ^2^Department of Infectious Disease and Tropical Medicine, Faculty of Public Health, Benghazi University, Benghazi, Libya; ^3^Neurological Rehabilitation Clinic, Stora Sköndal, Sköndal, Sweden; ^4^Department of Neurobiology, Care Sciences and Society, Karolinska Institutet, Stockholm, Sweden; ^5^Gottfries Clinic AB, Mölndal, Sweden; ^6^Division of Cell Biology, Department of Clinical and Experimental Medicine, Linköping University, Linköping, Sweden

**Keywords:** anti-herpesviral antibodies, human herpesviruses, HHV, Epstein-Barr virus, EBV, myalgic encephalomyelitis, ME/CFS, suspension multiplex immunoassay

## Abstract

Myalgic encephalomyelitis, also referred to as chronic fatigue syndrome (ME/CFS) is a debilitating disease characterized by myalgia and a sometimes severe limitation of physical activity and cognition. It is exacerbated by physical and mental activity. Its cause is unknown, but frequently starts with an infection. The eliciting infection (commonly infectious mononucleosis or an upper respiratory infection) can be more or less well diagnosed. Among the human herpesviruses (HHV-1-8), HHV-4 (Epstein-Barr virus; EBV), HHV-6 (including HHV-6A and HHV-6B), and HHV-7, have been implicated in the pathogenesis of ME/CFS. It was therefore logical to search for serological evidence of past herpesvirus infection/reactivation in several cohorts of ME/CFS patients (all diagnosed using the Canada criteria). Control samples were from Swedish blood donors. We used whole purified virus, recombinant proteins, and synthetic peptides as antigens in a suspension multiplex immunoassay (SMIA) for immunoglobulin G (IgG). The study on herpesviral peptides based on antigenicity with human sera yielded novel epitope information. Overall, IgG anti-herpes-viral reactivities of ME/CFS patients and controls did not show significant differences. However, the high precision and internally controlled format allowed us to observe minor relative differences between antibody reactivities of some herpesviral antigens in ME/CFS versus controls. ME/CFS samples reacted somewhat differently from controls with whole virus HHV-1 antigens and recombinant EBV EBNA6 and EA antigens. We conclude that ME/CFS samples had similar levels of IgG reactivity as blood donor samples with HHV-1-7 antigens. The subtle serological differences should not be over-interpreted, but they may indicate that the immune system of some ME/CFS patients interact with the ubiquitous herpesviruses in a way different from that of healthy controls.

## Introduction

Myalgic encephalomyelitis, also referred to as chronic fatigue syndrome (ME/CFS), is a common syndrome which includes post-exertional malaise (PEM), brain fog, unrefreshing sleep, hemodynamic abnormality, myalgia, and headache. Although diagnostic criteria gradually have become stricter, there is still some heterogeneity of symptoms as well as severity. Its cause is unknown, but one hypothesis is that it is an autoimmune disease (1). ME/CFS starts with an infection in approximately 70% of cases (2–10). The eliciting infection may be infectious mononucleosis (IM), often caused by Epstein-Barr virus (EBV), or an upper respiratory infection. An aberrant serological pattern of reactivity with individual EBV antigens has been found in some (11–21), but not in other (22–24) investigations. Besides EBV (9, 25) other herpesviruses that have been tentatively implicated in the pathogenesis, based on serology or nucleic acid detection, are human herpesvirus 6 [HHV-6A (26, 27), varicella-zoster virus (VZV) (28), cytomegalovirus (CMV), see e.g., (29), and HHV-7] (26). Although a direct causative effect of a herpesvirus may be unlikely (29), one or several of them may be involved in combinatorial pathogenic effects. A large portion of herpesviral genomes encode proteins which are specialized for immune evasion. The main target cells of herpesviruses belong to the immune (HHV-4, HHV-5, HHV-6A, HHV-7, and HHV-8) or nervous (HHV-1, HHV-2, HHV-3, HHV-6A, and HHV-7) systems. EBV can transactivate promoters involved in autoimmunity (30). The aim of this investigation was to search for serological evidence for or against an involvement of a herpesvirus, either as a trigger, or as a chronically active infection, in ME/CFS. It was also part of a search for biomarkers for ME/CFS, in several cohorts of ME/CFS patients. In the process, novel epitope information was revealed. IgG antibodies to a diverse set of herpesviral (HHV-1-HHV-7) antigens were analyzed in a suspension multiplex immunoassay (SMIA). The multiplex format allowed internally controlled measurements, increasing the precision of serological comparisons.

Specifically, we addressed the following questions, using SMIA: Can antibodies against synthetic herpesviral peptides, useful for serological differentiation of ME/CFS from blood donors (BD), be found? Can the frequency of seropositivity with HHV1-7 indicate if infection with a certain herpesvirus is more or less common among ME/CFS patients compared to controls? Can differences between ME/CFS and control samples, in degree of seroreactivity with HHV1-7 antigens be detected? Can previous reports of aberrant EBV viral capsid antigen (VCA), early antigen D (EA-D), Epstein-Barr virus nuclear antigen 1 (EBNA1), and EBNA6 antibody formation in ME/CFS be corroborated? Can previously found distinct EBNA1 peptide antibody patterns reported in multiple sclerosis (MS) also be found in ME/CFS sera?

## Materials and Methods

### Antigens

The following purified whole virus preparations were used as lysates in Triton X100 (purchased from Advanced Biotechnologies, Eldersburg, MD): Human herpesvirus type 1 (HHV-1; strain MacIntyre, purified virus lysate, catalog #10-145-000), HHV-2 (Strain G, purified virus lysate, #10-146-000), HHV-3 (VZV; strain Rod, purified virus lysate, #10-282-500), HHV-4 (EBV; strain B95-8, #10-147-000), HHV-5 (CMV; strain AD-169, #10-144-100), and HHV-6a (GS strain, #10-241-500).

Proteins from EBV were: gp125 (BALF4, the EBV counterpart of the gB protein, a VCA component) purified from infected cell cultures (East Coast Bio, North Berwick, ME, #EV045-7.5X). Recombinant proteins were p18 (BFRF3, #EBV-273, also a VCA component, amino acids 1-119), EBNA1 (#EBV-276, amino acids 408-641), and EA (type D, BMRF1, #EBV-272-a, amino acids 306-390), all from Prospec Bio, Rehovot, Israel. A non-herpesviral control antigen, *Haemophilus influenzae* vaccine (polysaccharide conjugated to tetanus toxoid, ACT-Hib, Sanofi Pasteur, Lyon, France), was included as a control in some of the SMIA tests.

Sequences of the synthetic peptides evaluated for this project are given in Table S1 in Supplementary Materials. They had an N-terminal spacer with the structure NH_2_-PEG_6_-His_6_-PEG_6_, where PEG_6_ denotes hexa-polyethylene glycol and His_6_ a hexahistidine antigen tag used for assessment of coupling efficiency.

The peptides were mostly 30 amino acids long. Their sequence was based on information from the Immune epitope database (IEDB), epitope information from the literature, and prediction programs at the IEDB site. All synthetic peptides were purchased from Xaia AB, Gothenburg, Sweden.

### Patient Samples

Several cohorts of samples from ME/CFS patients were included. All ME/CFS samples were diagnosed according to the Canada criteria (31, 32). The ME/CFS cohort 1 samples were collected at the Gottfries Clinic in Gothenburg during 2009, under ethical permission Dnr 680-09, 960-12 granted by the ethical commission of the University of Gothenburg. Likewise, ME/CFS cohort 2 was collected at the Gottfries clinic during 2007, under permission Dnr 806-11, 029-13. Cohort 3 (Stora Sköndal, *n* = 37) was collected during 2017 and 2018 with permission from the regional ethics committee in Stockholm 2016/1230-31/4.

#### Properties of Cohort 1 (Gottfries Clinic, Mölndal)

ME/CFS diagnosis alone; *N* = 46 (F = 34, M = 12). Mean age 45.8 yrs ± 9.2 (SD) years. Disease duration average 11.7 ± 7.7 yrs. Severity average 40.0 ± 9.1 (Fibrofatigue sum score range of scores 0–72) (33). Fibromyalgia (FM) diagnosis; *N* = 11 (F = 8, M = 3). Mean age 46.8 ± 10.7 yrs. Disease duration average 14.4 ± 10.1 yrs. Severity average 40.0 ± 13.5 (Fibrofatigue sum score). FM+ME/CFS diagnosis; *N* = 17 (F = 14, M = 3). Mean age 44.5 ± 9.7 yrs. Disease duration average 11.7 ± 7.7 yrs. Severity average 40.0 ± 9.1 (Fibrofatigue sum score). ME/CFS +Irritable Bowel Syndrome (IBS) IBS diagnosis; *N* = 2 (F = 2, M = 0). Mean age 44.5 ± 0.5 yrs. Disease duration average 13.5 ± 2.5 yrs. Severity average 47.5 ± 5.5 (Fibrofatigue sum score). To gain statistical power, categories containing ME/CFS patients (the first and last two categories) were here summarized as “All ME/CFS”, *N* = 65 (F = 50, M = 15). Mean age 45.4 ± 9.1 yrs. Disease duration average 11.0 ± 8.1 years. Severity average 40.6 ± 8.5 (Fibrofatigue sum score).

In the clinical investigation of the 5 patients with ME/CFS that were seronegative for EBV the clinician who made the diagnosis did not find any divergent symptoms, when comparing them with the main group. Two of them had an acute and 3 a slow onset. The patients were rated by the FibroFatigue scale (33). The scale has a total variance of 0–72. The 5 patients varied between 29 and 49. The one with the score 49 who was the most severely ill, had a slow onset and had not suffered from many infections. Likewise, the 3 EBV seronegative patients from cohort 3 did not differ from the total 37 patients in the cohort.

#### Properties of Cohort 2 (Gottfries Clinic, Mölndal)

ME/CFS diagnosis *n* = 61 (F = 51, M = 10). Mean age 46.9 ± 11.0 yrs, Disease duration 8.6 ± 10.0 yrs. Severity average 35.5 ± 7.8 (Fibrofatigue sum score).

#### Properties of Cohort 3 (Stora Sköndal)

ME/CFS diagnosis; *n* = 37 (F = 26, M = 11). Age average 42 ± 12 yrs. Disease duration 9 ± 5 yrs. Severity (work disability 100%): 26/37 (70%). Infectious onset: 30/37 (81%).

#### Anonymous Blood Donor Sera

Anonymous blood donor sera from the Uppsala Academic Hospital were used as negative controls. They were all used with informed consent according to the Swedish Biobank law (SFS 2002:297) which allows diagnostic patient samples to be used for similar purposes as the original sampling purpose. The 103 sera submitted to the routine clinical microbiology laboratory in Uppsala, for EBV diagnostic investigation collected during 2012 were kindly provided by Dr. Kåre Bondeson. They were tested anonymously. A permit (Ups 01-367) from the IRB of Uppsala University Hospital was obtained.

### Suspension Multiplex Immunoassay (SMIA)

Antigens were coupled to carboxylated differentially color-marked magnetic microspheres (MagPlex-C microspheres, Luminex Corp, Austin, TX) using carbodiimide, essentially as described (34, 35). Briefly, 100 μl of the stock microsphere solution (1.25 × 10^6^ beads) were coupled with 10 μg of whole virus, recombinant protein antigen or 50 μg of synthetic peptide antigen. After the coupling, beads were incubated with 0.5 ml PBS containing 0.05% (v/v) Tween20 and 50 mM Tris (PBS-T) in the dark for 15 min on a rocking mixer at room temperature, to block unreacted carboxyl groups with primary amines. The beads were then washed once with 0.5 ml StabilGuard (SurModics, Eden Prairie, MN, #SG01-1000) using a magnetic separator. The bead pellet was finally resuspended in 400 μl StabilGuard. We simultaneously tested 3 to 49 antigens at a time.

**Figure 1:**
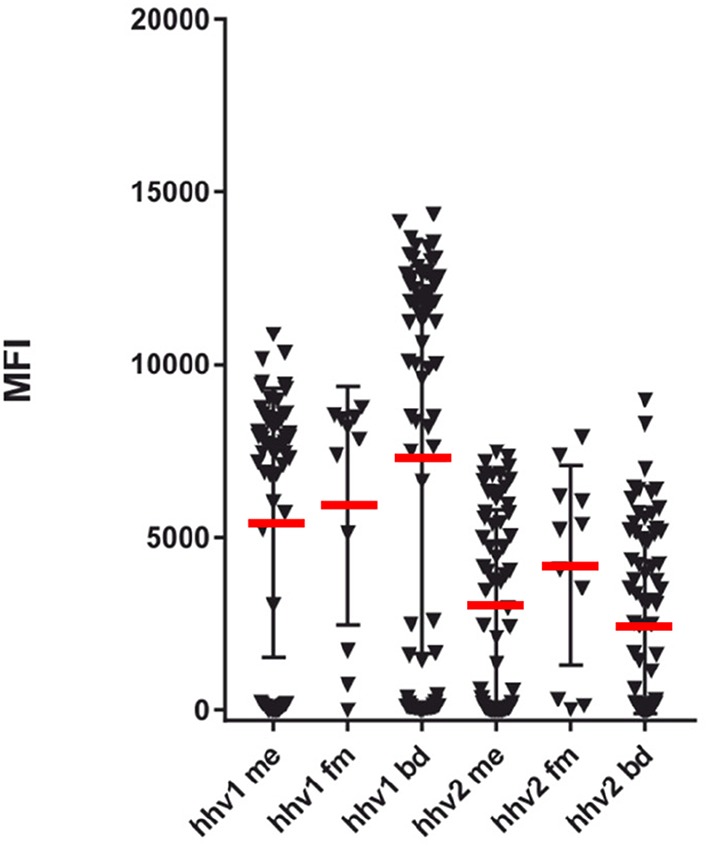
SMIA (lgG) of whole purified herpes simplex viral antigens (HHV-1 and HHV-2) with ME/CFS samples (Cohort 1, ME/CFS, *n* = 65 and FM, *n* = 11) and blood donor controls (BD; one cohort, *n* = 76). Reactivities (Median fluorescent intensity, MFI) in SMIA. me, ME/CFS patients; fm, fibromyalgia patients; bd, blood donors. Statistics by Fisher exact test.

**Figure 2:**
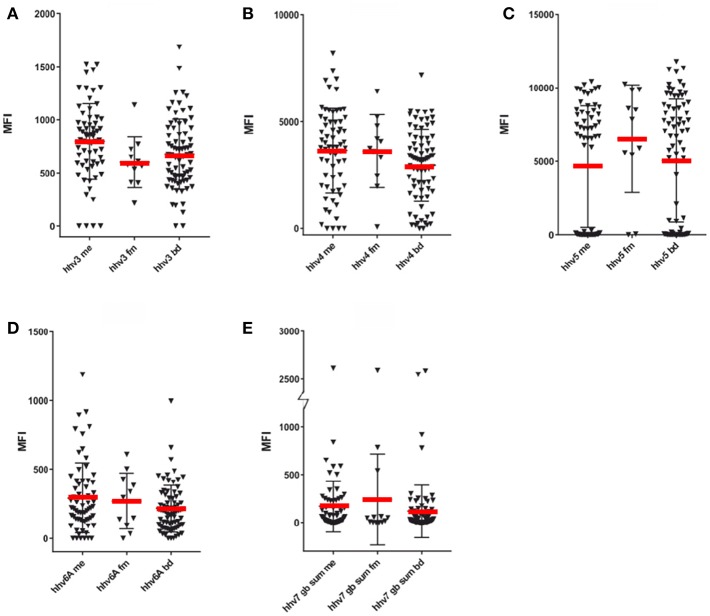
SMIA of whole purified virus lysate HHV-3- HHV-6 antigens and synthetic HHV-7 peptides with ME/CFS samples (Cohort 1, ME/CFS, *n* = 65 and FM, *n* = 11) and blood donor controls (BD; one cohort, *n* = 76). **(A)** HHV-3 (Varicella-Zoster virus), **(B)** HHV-4 (Epstein-Barr virus), **(C)** HHV-5 (Cytomegalovirus), **(D)** HHV-6A (Human herpesvirus 6A), **(E)** Sum of MFI with the 7 long HHV-7 (Human Herpesvirus 7) gB peptides detailed in Table S1. The Y axis was broken to be able to show both low and high reactivities.

**Figure 3:**
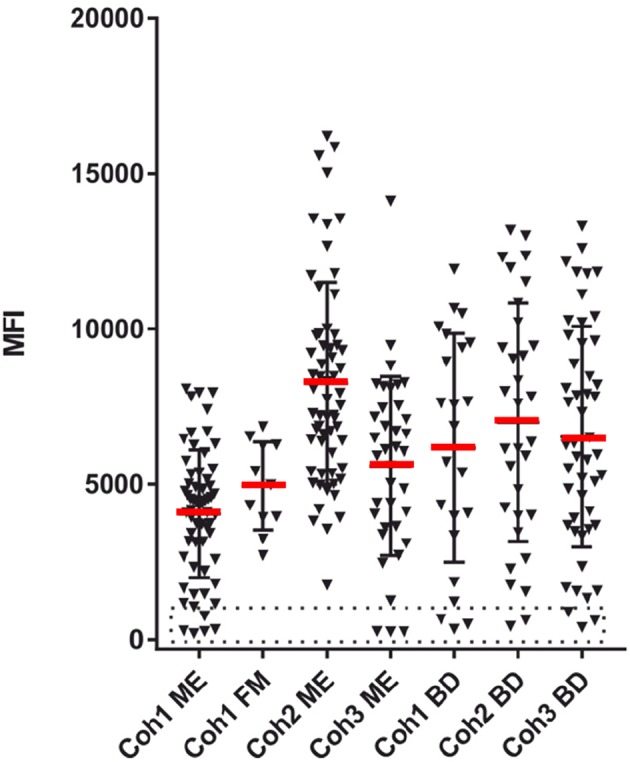
EBV seropositivity estimation. The sum of SMIA lgG MFI for VCA (p18 and gp125) and EBNA1 protein antigens from cohorts 1 (ME/CFS and FM), 2, and 3 (ME/CFS), and samples from three blood donor (BD) cohorts. The stippled area indicates absence of EBV antibodies.

**Figure 4:**
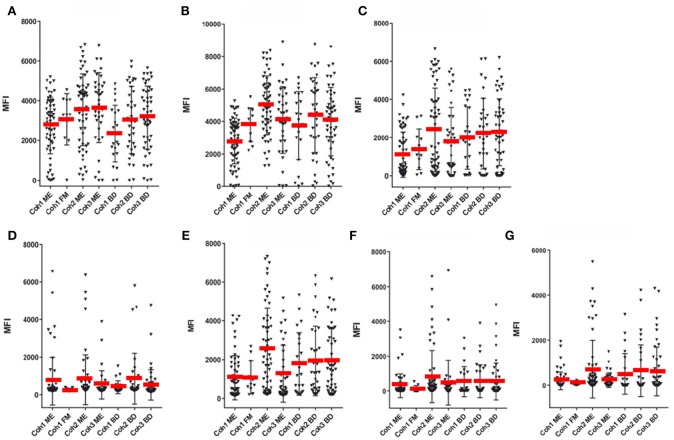
SMIA lgG with EBV antigens and cohort 1 (ME/CFS *n* = 61 and FM *n* = 11), cohort 2 (ME/CFS *n* = 61) and cohort 3 (ME/CFS *n* = 37), and three blood donor (BD) cohort samples (*n* = 46, 33, and 22, respectively). **(A)** gp125 (VCA) protein, **(B)** p18 (VCA) protein, **(C)** EBNA1 protein, **(D)** EA-D protein, **(E)** EBNA6 706–740 peptide. **(F)** EBNA1 381-410 peptide. **(G)** EBNA1 391-420 peptide. Abbreviations as in Figure 1.

**Figure 5:**
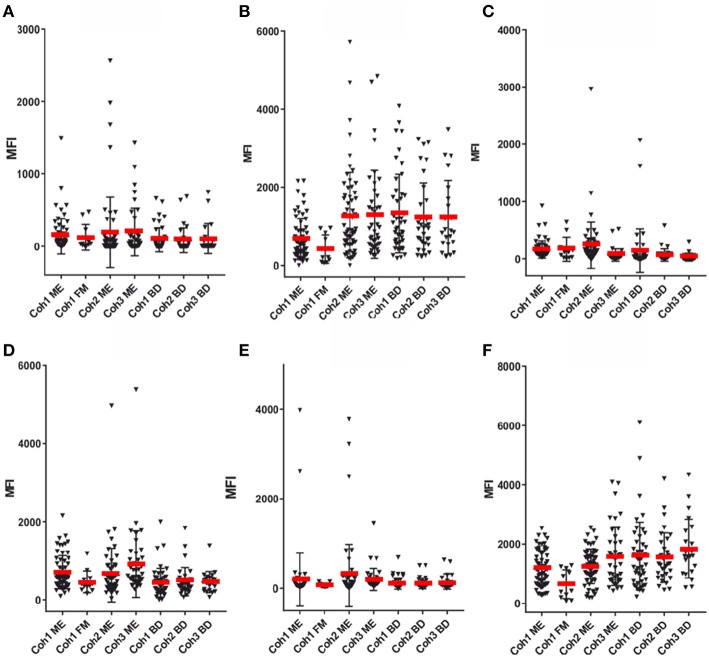
SMIA lgM for the same EBV antigens and sera as in Figure 4. **(A)** gp125 (VCA) protein, **(B)** p18 (VCA) protein, **(C)** EBNA1 protein, **(D)** EA-D protein, **(E)** EBNA6 706-740 peptide. **(F)** LMP2A 468-497 peptide. For abbreviations see legend of Figure 1.

**Figure 6:**
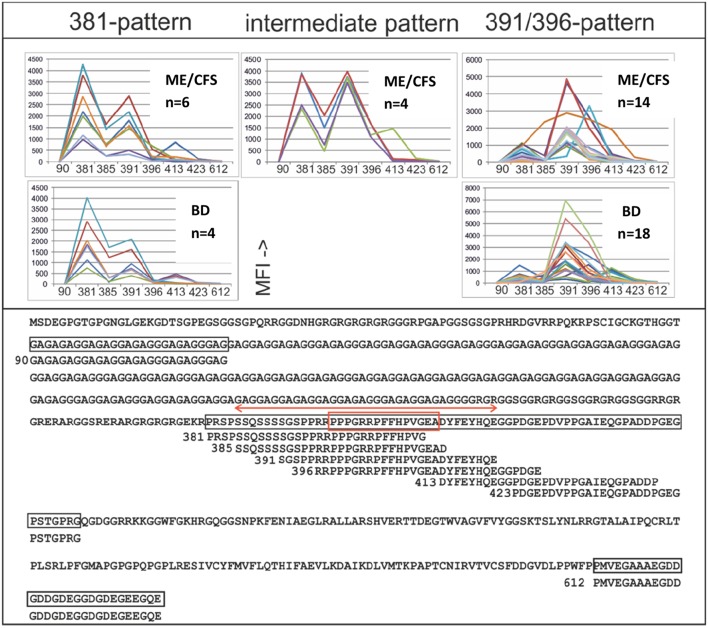
IgG epitope mapping of EBNA1 using overlapping and non-overlapping synthetic peptides, with ME/CFS cohort 1 and blood donor sera. Lower panel: The entire EBNA1 sequence (YP 401677), from EBV strain B95-8, and synthetic peptides derived from it, is shown. Peptides are identified by the sequence number of their first amino acid. The red double arrow shows a peptide which reportedly reacted preferentially with MS sera compared to controls (36). The red boxed sequence was reported to be frequently temporarily recognized by IgG after recent infectious mononucleosis and to cross-react with autoantigen Sm B′ (37). Upper panel: IgG reactivities (MFI) of sera which gave more than 1,000 MFI with any of the peptides. The three major peptide reaction patterns (381-; left, intermediate; middle and 391/396–pattern; right) are shown.

Serology was conducted using (a) 50 μl of serum diluted 1/20, final dilution of 1/40 (results shown in Initial peptide screening, Figures 1, 2), and (b) 50 μl of serum diluted 1/50, final dilution of 1/100 (results shown in Figures 3–**6**) in PBS, pH 7.4, containing 0.05% (v/v) Tween 20, 50 mM Tris and 2% (v/v) Prionex (Sigma-Aldrich, Saint Louis, MO, #81662) (PBS-TP) was added to wells of a round bottom 96-well microtiter plate (Greiner, Thermofisher, Carlsbad, CA, #104650) excluding the blank and controls. Fifty μl of a vortexed and sonicated bead mixture consisting of 25 beads/μl suspended in PBS-TP was then added to each well. The plate was then incubated in the dark with gentle rotation for 1 h at 37°C. Post incubation the wells were washed with PBS using a magnetic plate separator (Life technologies, Thermofisher, #A14179). Beads were resuspended in 50 μl of PBS-TP and 50 μl of biotinylated-protein G (Pierce, Thermofisher, article # 29988; 4 μg/ml of PBS-TP) in each well. For IgM antibody analysis, 50 μl of anti-human IgM (μ-chain specific) biotinylated antibodies produced in goat (Sigma B1265, Lot#SLBR6322V) was added into each well. The plate was then incubated for 30 min at 37°C in the dark with rotation. The plate was then incubated for 30 min at 37°C in the dark with rotation. After washing with PBS the beads were resuspended in 50 μl of PBS-TP followed by the addition of 50 μl of streptavidin-phycoerythrin (SA-PhE) (InVitrogen-ThermoFisher, article # S-866; 4 μg/ml in PBS-TP) in each well, and incubated for 15 min at 37°C in the dark with rotation. Beads were washed once with PBS before they were resuspended in 100 μl of PBS and analyzed in a Luminex-200 (Luminex Corp) instrument according to the instruction from the manufacturer. A minimum of 100 events for each bead number was set and the median fluorescence intensity (MFI) was calculated. Control beads were; a non-coupled (“naked” or “blank”) bead, and a bead coupled with hexahistidine (“His_6_“). The His_6_ bead allowed monitoring of coupling of the peptides to the magnetic beads using anti-hexahistidine antibodies (Antibodies online, Aachen, Germany, #ABIN100493). We did not observe false positive reactions due to anti-His_6_. One negative control, where PBS-TP instead of serum was added, was also used in all experiments. Reported MFI for an antigen was from the bead with the antigen minus the MFI of the “naked” bead. The MFI of the naked bead were 20-90 MFI.

### Comparative Serologies

HHV-1+2: HerpeSelect; Focus diagnostics (DiaSorin molecular, Cypress, CA) HHV-1 (catalog # EL0910G), and HHV-2 (# EL0920G) EIA, HHV-3: Enzygnost VZV IgG Siemens EIA (#PI-15-019, Siemens Healthcare diagnostics, Eschborn, Germany); HHV-4: Enzygnost EBNA IgG, Enzygnost EBV VCA IgG EIA (Siemens), HHV-5: CMV IgG Enzygnost EIA (#OWBA155, Siemens), and HHV-6: HHV-6 IgG EIA (#ODZ-235, Mobitec AG, Göttingen, Germany). These tests were performed according to the instructions of the manufacturer. There was no commercial counterpart for HHV-7 serology. Validations for HHV-4 whole virus, VCA and EBNA1 SMIAs are shown in Table S2, in Supplementary Materials. Other validations have been published (38, 39).

### Statistical Methods

The two-tailed Fisher exact and/or Mann-Whitney U tests were used.

## Results

### Validation of the Serological Procedures

The purpose of the present investigation was not to establish diagnostic techniques ready for routine use. Rather, the intention was to use as pure and antigenically active antigens as possible to represent a variety of herpesviral epitopes. Several of the whole virus SMIA components, for HHV-1, HHV-3, HHV-5, and HHV-6A, have previously been evaluated for correlation with commercial tests (38, 39). The HHV-2 whole virus SMIA component gave a concordance with a type-specific HHV-2 IgG test (Herpeselect 2) of 33%. HHV-1 and HHV-2 whole virus antigens cross-react extensively. The low concordance likely depends on cross-reactions from the more prevalent HHV-1. Validations for HHV-4 whole virus, VCA and EBNA1 SMIAs are shown in Table S2, in Supplementary Materials. Other validations have been published (38, 39). The HHV-4 single peptide VCA p18 and EBNA1, and recombinant VCA p18, VCA gp125, and EBNA1 were highly correlated with the Siemens VCA and EBNA1 comparative tests, respectively.

### Epitope Study Using Overlapping and Non-overlapping Synthetic Peptides

Synthetic 30mer peptides were evaluated for antigenicity against positive and negative control samples, samples from cohort 1 (*n* = 75) and blood donors (*n* = 75). The use of these two cohorts for epitope discovery was justified by the ubiquity of HHV-1–HHV-7 (25–98%) (40–43). Thus, it is likely that the sera contained antibodies to many of the studied herpesviruses. Using criteria detailed in the Supplementary Materials, a minority of the peptides proved to be frequently antigenic, indicating useful sensitivity. Specificity was indicated by correlation with results of comparative tests (Table S2). For EBV, the EBNA1 391–420 and VCA p18 119–148 peptides were chosen based on correlation with results of a commercial test and frequent antigenicity. These and other EBV peptides were run together with VCA and EBNA1 recombinant proteins. For EBNA6, two peptides which included the highly antigenic repeat region (44) were used (Table S1). Results with the longer peptide, which gave the strongest reactions (data not shown), are reported. Among the HHV-6 peptides, none were reactive frequently enough for selection. Among the HHV-7 peptides, the long peptides from HHV-7 gB were chosen as a set, letting the sum of their reactivity approximate HHV-7 antibody reactivity. Thus, synthetic peptides potentially useful for herpesvirus serology were defined.

### Antibodies to Whole Virus Preparations (HHV-1 to HHV-6A) and HHV-7 Peptides

As seen in Figure 1, the degree of HHV-1 reactivity of ME/CFS and FM patients were lower than those of blood donor controls. In contrast, the HHV-2 antigen reacted equally in the three categories. However, when the degree of reactivity (MFI) for ME/CFS and BD samples were compared using the Mann-Whitney U test, neither HHV-1, and HHV-2 antigens showed significant differences (*p* > 0.05).

The frequency of seropositivity for the tested herpesviruses can be seen in Figures 1, 2. Using an operational cut-off of 300 MFI for HHV-1-HHV-5 and of 100 MFI for HHV-6A and HHV7, the seropositivity rates for cohort 1 (ME/CFS and FM) and blood donors, respectively, are shown in Table 1. None of the seropositivity frequencies of ME/CFS vs. those of BD were significantly different (Fisher exact test; *p* >0.1). Thus, the herpesvirus particle and long gB peptide IgG assays did not show differences in degree of seropositivity between ME/CFS and BD.

**Table 1:** Seropositive frequencies of herpesviruses HHV-1 to HHV-7 in cohort 1.

**Herpesvirus**	**ME/CFS**	**Fibromyalgia**	**Blood donors**
HHV-1	44/65 (68%)	8/11 (73%)	54/76 (71%)
HHV-2	42/65 (65%)	10/11 (91%)	43/76 (57%)
HHV-3	58/65 (89%)	11/11 (100%)	70/76 (92%)
HHV-4	60/65 (92%)	10/11 (91%)	68/76 (89%)
HHV-5	37/65 (57%)	9/11 (82%)	51/76 (67%)
HHV-6A	54/65 (83%)	9/11 (82%)	54/76 (71%)
HHV-7	25/65 (38%)	3/11 (27%)	19/81 (23%)

*Operational cut-off of 300 MFI for HHV-1-5 and 100 MFI for HHV-6A and HHV-7*.

The anti-herpesviral IgG reactivities (MFI) can also be seen in Figures 1, 2. The reactivities to whole virus HHV-1-HHV-6, and long gB peptide was not significantly different between ME/CFS samples and controls using the Mann Whitney U test (*p* > 0.05).

### Antibodies to EBV Proteins and Synthetic Peptides

A total of 8 ME/CFS patients, 5 from cohort 1 and 3 from cohort 3, were EBV seronegative using the sum of VCA and EBNA1 as a criterion. However, cohort 1 was also tested with whole virus particle EBV antigen lysate. Of the 5 EBV seronegative cohort 1 samples seen in Figures 3, 4 reacted moderately strong with the whole EBV antigen lysate (data not shown). Thus, the exact extent of EBV seropositivity was not certain. A “gray zone” containing 4 of the 8 seronegative samples, possibly influenced by cross-reactions from the other herpesviruses, had to be declared.

Figures 3–5 show that none of the 5 EBV antigens (Figure 4) and 6 EBV antigens (Figure 5) used for IgG and IgM serology, respectively, gave significant differences between ME/CFS and BD samples. However, anti-EA IgM (Figure 5D), and anti-EBNA6 IgM (Figure 5E) showed a trend to react differently in ME/CFS samples versus BD control samples (*p* > 0.05, Fisher exact test) but did not reach statistical significance.

The LMP2A peptide gave a very low IgG reactivity (not shown), and also low IgM reactivity (Figure 5F).

### Results With Fibromyalgia Samples

A small number of FM samples were included in cohort 1. A greater number of FM samples would have been required to reach firm conclusions regarding differences in herpesviral antibody reactivities between ME/CFS and FM. FM MFI followed approximately those of ME/CFS for VCA and recombinant EBNA1 antigens, for both IgG and IgM. However, some differences were notable. FM sample IgG reactivities with EA-D and EBNA1 peptide 391–420 were low. The average EA-D IgG reactivity was not significantly different for the 11 FM samples compared to EA-D IgG for the 65 cohort 1 ME/CFS samples (Figure 4).

### Detailed Study of EBNA1 Epitopes

EBNA1 epitope mapping was conducted with a) cohort 1 samples and blood donor controls (Figure 6), and b) Twenty multiple sclerosis samples kindly provided by Dr. Jan Fagius, Department of Neurology, Uppsala, Sweden. Two major peptide IgG recognition patterns (Figure 6) were seen. One pattern featured a major reaction (>1,000 MFI) with EBNA1 381–410 and less with the other overlapping peptides. Another prevalent pattern was a strong reaction (>1,000 MFI) with either or both of peptides EBNA1 391-420 and EBNA1 396-425 (the “391/396 peptides”), and weaker reactions with the other peptides. As depicted in Figure 6, 6/65 ME/CFS and 4/76 BD samples gave a 381-pattern, 4/65 ME/CFS and 0/76 BD an intermediate, and 14/65 ME/CFS, and 18/76 BD a 391/396-pattern. The difference between 6/65 and 14/65 for ME/CFS and 4/76 and 18/76 for BD was not significant. The 391/396 peptides contain epitopes reported to be selectively recognized by certain MS sera (36), and an epitope which is cross-reactive with an auto-epitope (37).

Although MS was not a major part of this investigation, 20 MS sera were tested together with 163 ME/CFS and 79 BD sera against the 381, 385, 391, and 396 peptides for IgG and IgM reactivity. Of the 20 MS sera 3 had a 381, 2 a 391/396 and 1 an intermediary IgG pattern. However, the MS sera were only weakly IgM reactive with peptides 391 and 396, while ME/CFS and BD sera were strongly reactive to these peptides. Thus, the reported tendency of peptides from the 391/396 sequence to react differentially with IgG in MS patients (36) could not be corroborated. Neither was there a separate IgG reactivity pattern of ME/CFS sera with the four peptides. However, the 391 and 396 peptides reacted differentially, but not significantly with IgM in the small number of MS sera compared to ME/CFS and BD sera, warranting further studies.

## Discussion

The aim of this study was twofold; first to repeat and extend previously reported serological herpesvirus results with ME/CFS sera; and second to obtain leads for further research on ME/CFS pathogenesis and biomarkers. The aim was not to validate new herpesvirus serologies suitable for routine diagnostic use. Rather, we wanted to search for evidence of past exposure to a herpesvirus (seropositivity) and increased antibody levels indicative of viral (re)activation. Multiplex serology, like SMIA, can provide much data in a short time. It is a precise tool, where highly reproducible parameters can be derived from relations between reactions to several antigens because each MFI value is the median of 100 measurements and measurements are made in the same reaction volume, eliminating pipetting error. Nevertheless, like in all serologies, interpretation can be challenging.

### HHV-1 and HHV-2

The seroprevalence of HHV-1 and HHV-2 in Swedish adults is reported to be 80% and 13%, respectively (45). Our values were 68–71 and 57–65%, respectively. HHV-1 and HHV-2 cross-react extensively (46) and exact seroprevalence measurements are not possible when whole virus preparations are used.

### HHV-3 (VZV)

An involvement of VZV in ME/CFS was inferred (28). However, neither seropositivity rate nor degree of reactivity differed significantly between ME/CFS and healthy controls.

### HHV-4 (EBV)

EBV is an established trigger of ME/CFS, see e.g., (1). It is therefore of interest to search for EBV negative ME/CFS cases. We found a few. They did not differ appreciably from the other ME/CFS cases. Thus, it is unlikely that EBV infection is obligatory for development of ME/CFS. EBV serology is a complicated matter, calling for cautious interpretation, see e.g. (47).

A long trail of studies on EBV serology in CFS [using the “Fukuda criteria” (48)] and ME/CFS [using “Canada criteria” (31, 32)] reported a higher frequency of the combination of high VCA and lower EBNA1 IgG reactivities (titers) in ME/CFS sera than in control sera (11–21). In early papers on CFS, it was noted that EBNA1 antibody levels tended to be low relative to the levels of viral capsid antigens (VCA, a term stemming from the time of immunofluorescence diagnosis, including capsid antigens p18 and p23, and glycoprotein gp125) in sera from CFS patients relative to those of healthy controls (49). Such a pattern can be thought of as an extension of the acute period (0–3 months post start of infection) of primary EBV infection, where VCA and EA IgG and IgM dominate and EBNA1 IgG remains negative. However, we did not see higher levels of VCA IgG or IgM, or lower EBNA1 IgG antibodies in any of the three ME cohorts, compared with BD controls. A reason may be that the recombinant EBNA1 protein, covering amino acids 408-641, did not include the long glycine-alanine repeat region (amino acids 90-327). However, our EBNA1 peptide 90-120 consisted of Gly-Ala repeats, but was not frequently antigenic in our analyses. Reasons for our inability to repeat previous results could be methodological differences, or our use of the more strict Canada inclusion criteria (31, 32). We did not see an increased VCA IgG reactivity over EBNA1 in any of the three ME/CFS cohorts, compared with BD controls.

There is precedence for differential reactivity to these EBV antigens in the autoimmune disorders systemic lupus erythematosus (SLE) (50, 51) and multiple sclerosis (MS) (52, 53). In SLE, an increase in VCA IgG, VCA IgM, EA-D IgG and high (54) or low (55) EBNA1 IgG was reported. In MS, high EBNA1 antibodies, mainly to the glycine-alanine repeat, were reported (36, 56–58). A special and rather rare case is the so-called chronic active EBV syndrome, a potentially life-threatening condition with high EBV DNA and VCA IgG levels, which occurs in certain immunodeficiencies (13, 59–64). Our study with overlapping synthetic peptides identified two major IgG patterns to the immunodominant portion of EBNA1 (amino acids 381-420), situated after the long glycine-alanine repeat (aa 91-327). One pattern mainly involved reactivity with the 381-410, the other with the 391-420 peptide. However, the frequencies of the two patterns were not significantly different in ME/CFS and BD controls.

EA-D antibodies, especially of the IgA class, are associated with certain diseases. Most studied is the correlation of IgA anti-EA-D with nasopharyngeal carcinoma (65). In the present study the EA-D IgM levels were higher, but not significantly, in ME/CFS compared to BD controls in cohorts 1, 2, and 3.

IgG antibodies measured using a synthetic peptide from the repeat region of EBNA6 had a similar kinetic during primary EBV infection as EBNA1 (44), i.e., becoming positive late during primary infection. A peptide containing this sequence (QPAPQAPYQGYQEPP; EBNA6, aa 740-754 according to UniprotKB P03204), as well as a recombinant EBNA6 protein, were reported by Loebl et al. to give a somewhat stronger reactivity with ME sera than control sera in an ELISA (66). In our hands, with a covalently coupled EBNA6 peptide, IgG reactivities were not significantly different between the ME/CFS samples and healthy control samples. Our EBNA6 peptide was longer and overlapped with 14 of the 15 amino acids of the Loebl et al. peptide. However, our peptide did react differentially, measured as an EBNA6/LMP2A ratio, with a subset of ME/CFS sera in IgM. This should be further investigated with more EBNA and VCA antigens and more samples.

There is growing evidence for the existence of pathogenesis-related EBV variants (67). Further work may give a consensus on EBV serology in ME/CFS patients and perhaps a correlation between serological features and EBV sequence.

### HHV-5 (CMV)

The CMV seroprevalence is around 50% in the Swedish population (68). CMV reactivation is common in immunosuppressed patients and pregnant women. In our study, there was, however, neither a difference in IgG seropositivity nor in IgG reactivity between ME/CFS samples (ME/CFS cohort 1) and healthy controls.

### HHV-6A and HHV-6B

HHV-6B gives a common early childhood infection, exanthema subitum, while older children and adults can get an often asymptomatic HHV-6A infection (69, 70). HHV-6A and HHV-6B give extensive serological cross reactions, and also cross-react with HHV-7 (71). HHV-6 IgG seropositivity in Swedish adults is at least 85% (72), like in other parts of the world (43, 73). Antibody titres to HHV-6 and HHV-7 were reported to be higher in ME/CFS patients than in controls (16, 18).

In our study, there was, however, neither a difference in IgG seropositivity rate nor in IgG reactivity for HHV-6 between ME/CFS samples and healthy controls. However, our HHV-6 serology gave seropositivity rates for HHV-6 of 70–80%. The assay obviously did not have an optimal sensitivity. We were not helped by the large number of synthetic peptides whose antigenicity we explored. The whole virus antigen lysate contained a broad representation of structural antigens, and thus can be considered a representative HHV-6 serology. We used it only as an indicator of possible differences in exposure or reactivation of the HHV-6A and HHV-6B viruses. With this limitation in mind, we did not see significant IgG reactivity differences for HHV-6 between cohort 1 ME/CFS patients and blood donor controls.

### HHV-7

Although not extensively studied, HHV-7 seroprevalence seems to be very high in Sweden, see e.g., (68), and elsewhere (41, 43). Exact estimates are hard because HHV-7 cross-reacts with both HHV-6A and 6B. Very few of the many HHV-7 peptides which we evaluated were sufficiently antigenic in SMIA with ME/CFS and BD samples. Although the 100 amino acid long gB peptides (whose IgG reactivities were summed as a proxy for HHV-7 IgG) were more frequently antigenic than the 30 amino acid peptides from HHV-7 they probably reacted only with a subset of HHV-7 antibody positive sera. Our HHV-7 seropositivity rate was 20-40%. Like for HHV-6A, our HHV-7 assay thus had a suboptimal sensitivity. However, there was neither a difference in IgG seropositivity rate nor in degree of IgG reactivity for HHV-7 between ME/CFS samples and healthy controls.

### Fibromyalgia

Fibromyalgia is a condition which overlaps ME/CFS. A few FM samples from FM patients, which fulfilled the FM ACR criteria (74), but did not fulfill the Canada criteria for ME/CFS, were included in cohort 1. With some herpesviral antigens, the FM samples behaved similar to the ME/CFS samples from cohort 1. With other antigens, there seemed to be a difference, although the low sample number (*n* = 11) precluded obtaining a statistical significance. It would be interesting to see if these trends could be corroborated in a study with more FM samples.

## Conclusions

HHV-1–HHV-7 are ubiquitous and have evolved sophisticated means of interaction with the human host. In essence, they can be regarded as pervasive environmentally acquired modulators of human immunity and other mechanisms, although the extent of this influence is uncertain. Our survey did not reveal infections with any of these herpesviruses to be more common or intense in ME/CFS patients compared to controls. However, we observed minor relative differences in antibody reactivities in ME/CFS samples versus controls with whole virus HHV-1 antigens and recombinant EBV EBNA6 and EA antigens. We conclude that these serological differences indicate that the immune system of some ME/CFS patients may interact with the ubiquitous herpesviruses in a way different from that of healthy controls and constitute a basis for further investigation on ME/CFS.

## Data Availability

All datasets generated for this study are included in the manuscript and/or the Supplementary Files.

## Ethics Statement

The ME/CFS cohort 1 samples were collected at the Gottfries Clinic in Gothenburg during 2009, under ethical permission Dnr 680-09, 960-12 granted by the ethical commission of the University of Gothenburg. Likewise, ME/CFS cohort 2 was collected at the Gottfries clinic during 2007, under permission Dnr 806-11, 029-13. Cohort 3 (Stora Sköndal, *n* = 37) was collected during 2017 and 2018 with permission from the regional ethics committee in Stockholm 2016/1230-31/4.

## Author Contributions

JB: conceived of the paper and wrote most of it. AB-W, AE, PJ, OZ, and C-GG: resources. JB and AR: funding acquisition. JB, MR, and AE: investigation. JB and AR: supervision. JB, MR, AB-W, AE, PJ, OZ, AR, and C-GG: writing, reviewing, and editing.

### Conflict of Interest Statement

The authors declare that the research was conducted in the absence of any commercial or financial relationships that could be construed as a potential conflict of interest.
