# Supplementary material for: Antibodies to Human Herpesviruses in Myalgic Encephalomyelitis/Chronic Fatigue Syndrome Patients
Source: Front Immunol. 2019 Aug 14;10:1946. doi: 10.3389/fimmu.2019.01946 (PMC6702656; doi:10.3389/fimmu.2019.01946)
Supplement: Supplementary file 1 [file Data_Sheet_1.PDF]

Supplementary Material

Table S1. Human herpesvirus peptides evaluated for this paper. All peptides had an N-terminal spacer, as described in the main text.

Virus	Protein, with amino acid positions	Accession number	Sequence
EBV1	EBNA1 382-411	YP 401677	PRSPSSQSSSSGSPPRRPPPGRRPFFHPVG***
EBV1	EBNA1 385-414	YP 401677	SSQSSSSGSPPRRPPPGRRPFFHPVGEADY*
EBV1	EBNA1 391-420<-	YP 401677	SGSPPRRPPPGRRPFFHPVGEADYFEYHQE****
EBV1	EBNA1 396-426	YP 401677	RRPPPGRRPFFHPVGEADYFEYHQEGGPDGE**
EBV2	EBNA1 401-430	CEQ43463	GRRPFFHPVAEADYFEYHQEGGPDGEPDMP**
EBV1	EBNA1 413-442	YP_401677	DYFEYHQEGGPDGEPDVPPGAIEQGPADDP*
EBV1	EBNA1 423-452	APD76303	PDGEPDVPPGAIEQGPADDPGEGPSTGPRG*
EBV2	EBNA2 170-199	YP_001129441	ALGHTLQPTPPPRPTLPQPRIPLIIPPRHT
EBV1	EBNA2 206-235	YP_401644	LPPATLTVPPRPTRPTTLPPTPLLTVLQRP*
EBV2	EBNA2 425-454	YP_001129441	WEGIFETTESHSSDEENVGGPSKRPRTSTQ
EBV1	EBNA2 458-478	YP_401644	WDYIFETTESPSSDEDYVEGPSKRPRPSIQ
EBV1	EBNA6/3C 706-740<-	ALV82829	**PAPQAPYQGYQEPPAPQAPY**QGYQEPPAPQAPYQG***
EBV1	EBNA6/3C 701-730<-	ALV82897	DVAAQ**PAPQAPYQGYQEPPAPQAPY**QGYQE**
EBV1	P18 119-148(VCA)<-	YP_401651	STAVAQSATPSVSSSISSLRAATSGATAAA***
EBV1	P18 147-176(VCA)	YP_401651	AASAAAAVDTGSGGGGQPQDTAPRGARKKQ
EBV1	LMP1 188-217	YP_401722	GQRHSDEHHHDDSLPHPQQATDDSGHESDS
EBV2	LMP2a 468-497<-	YP_401631	GVIRCCRYCCYYCLTLESEERPPTPYRNTV*
EBV1	gB 828-857	YP_401713	ARDRFPGLRRRRYHDPETAAALLGEAETEF
EBV1	ZEBRA 1-30	YP_401673	MMDPNSTSEDVKFTPDPYQVPFVQAFDQAT*
EBV1	ZEBRA 142-171	YP_401673	ADIGVPQPAPVAAPARRTRKPQQPESLEEC
EBV1	ZEBRA 165-184	YP_401673	PESLEECDSELEIKRYKNRVASRKCRAKFK*

HHV6A	gB 324-353	NP_042932	PKESLNLTDPKQTCIKNEFEKIINEVYMSD
HHV6A	gB 335-364	NP_042932	QTCIKNEFEKIINEVYMSDYNDTYSMNGSY
HHV6A	gB 348-377	NP_042932	EVYMSDYNDTYSMNGSYQIFKTTGDLILIW
HHV6A	gB 360-389	NP_042932	MNGSYQIFKTTGDLILIWQPLVQKSLMFLE
HHV6A	gB 372-401	NP_042932	DLILIWQPLVQKSLMFLEQGSEKIRRRRDV*
HHV6A	gB 384-413	NP_042932	SLMFLEQGSEKIRRRRDVGDVKSRHDILYV*
HHV6B	gB 794-823	NP_050229	DESYRRKPSSSESHASKPSLIDRIRYRGYK
HHV6B	gB 801-830	NP_050229	PSSSESHASKPSLIDRIRYRGYKSVNVEEA
HHV6B	gH 327-356	NP_050229	SIDSFVNRCVNVSEGTIQYPKMKEFLKYEP
HHV6B	gH 503-532	NP_050229	DMMEMLSVYRPPDMARVAAIQCLSPSEPAA
HHV6A	gH 617-646	AVK93385	QYIYIKNIDELKTLTDPNNNLLVPNTRTHY
HHV6A	gO 31-60 (62-91)	NP_042940	DPLEAFKTVNRHNWSDEQREHFYDLRNLYT
HHV6A	U24 1-30	NP_042917	MDPPRTPPPSYSEVLMMDVMCGQVSPHVIN
HHV6B	U24 1-30	NP_050204	MDRPRTPPPSYSEVLMMDVMYGQVSPHASN
HHV7	U24 _1-30	YP_073779	MTHETPPPSYNDVMLQMFHDHSVFLHQENL
HHV7	gB 2-31	YP_073779	KILFLSVFITFSLQLSLQTEADFVMTGHNQ
HHV7	gB 125-154	YP_073779	EARCLSSISVKRSEEEEYVAYHKDEYVNKT
HHV7	gB 206-235	YP_073779	PFDFFALSTGETVEGSPFYNGINSKTFNEP
HHV7	gB 404-433	YP_073779	KDIVYVQLQYLYDTLKDYINTALGKLAEAW
HHV7	gB 692-721	YP_073779	LTLGVIGLVIFLFLRHKRLAQTPIDILFPY
HHV7	gB 735-764	YP_073779	SVQAQVKEPLDSSPPYLKTNKDTEPQGDDI
HHV7	gB 793-822	YP_073779	EIAEAKKSQRPSLLERIQYRGYQKLSTEEL
HHV7	U14 155-184	YP_073779	ALKMIYRAGNSFDNQPDNDIESYNEKLKIY
HHV7	U14_354-383	YP_073779	VTFILTSSKESDDEYDEDKPPRQVDPDRVD
HHV7	U14 480-509	YP_073779	VLNISRPGSTTPSGNSARYGNNTPRSITPV
HHV7	U14 567-596	YP_074779	ERLSTNSPISINGNTPRQQSHGDNEIQTID

HHV7	U14 580-609	YP_074779	NTPRQQSHGDNEIQTIDSTDEDSMNAPQSP
HHV7	U14 619-648	YP_074779	VSTDDQLLHSPTNSPFNLFDSVSEMQEDTE
HHV7	U11 267-296	YP_074779	ITKSLELHALPVKSTWDDRVKFTPEPIQTF
HHV7	U11 702-731	YP_074779	LLTGKETQNTIFGASKAQENGDKDLIDLEN
HHV7	U11 726-755	YP_074779	LIDLENSVQKDDDIVNKLVSHLTHSEEDVV
HHV7	gp65 229-258	YP_074779	IPKVNMTSEKPLYDACCPDKNKSRENTTVA
HHV7	gp65 239-268	YP_074779	PLYDACCPDKNKSRENTTYAWRWSEHPWTE
HHV7	gp65 249-278	YP_073779	NKSRENTTYAWRWSEHPWTETTIEPWRDID
HHV7	gp65 439-468	YP_073779	SLNAQLCNSGNITQAFNNTVSEKLQNVMGG
HHV7	gB 161-260	YP_073779	NFKSDTVRRYITTKEPFLRNGPLWFYSTSTSINCIVTD CIAKTKYPFDFFALSTGETVEGSPFYNGINSKTFNEPT EKILFRNNY
HHV7	gB 241-340	YP_073779	FRNNYTMLKTFDDGSKGNFVTLTKMAFLEKGNTIFSWE VQNEESSICLLKHWMTIPHALRAENANSFHFIAQELTA SFVTGKSNYTLSDSKYNCINSNYT
HHV7	gB_321-420	YP_073779	GKSNYTLSDSKYNCINSNYTSILDEIYQTQYNNSHDKN GSYEIFKTEGDLILIWQPLIQRKLTVLENFSNASRKRR KRELETNKDIVYVQLQYLYDTLKD**
HHV7	gB_401-500	YP_073779	ETNKDIVYVQLQYLYDTLKDYINTALGKLAEAWCLNQK RTITVLHELSKISPSGIISAVYGKPMSAKLIGDVLAVS KCIEVNQTSVQLHKSMRLTKDSSY*
HHV7	gB_481-580	YP_073779	VNQTSVQLHKSMRLTKDSSYDALRCYSRPLLTYSFANS SKETYLGQLGLDNEILLGNHRTEECEQSNTKIFLSGKF AHIFKDYTYVNSSLITEIEALDAF
HHV7	gB_561-660	YP_073779	KDYTYVNSSLITEIEALDAFVDLNIDPLENADFTLLEL YTKDELSKANVFDLETILREYNSYKSALHHIETKIATV TPTYIGGIDTFFKGLGALGLGLGA
HHV7	gB_641-740	YP_073779	IGGIDTFFKGLGALGLGLGAVLGVTAGALGDVVNGVFS FLKNPFGGALTILLTLGVIGLVIFLFLRHKRLAQTPID ILFPYTSKSTNSVLQATQSVQAQV
HHV7	gB_721-822	YP_073779	YTSKSTNSVLQATQSVQAQVKEPLDSSPPYLKTNKDTE PQGDDITHTNEYSQVEALKMLKAIKLLDESYKKAEIAE AKKSQRPSLLERIQYRGYQKLSTEEL**

Footnotes:

1. Antigenicity, frequency of reactivity with blood donor (n=16) and ME blood samples (n=75, cohort 1) is approximated as asterisks. * denotes antigenicity of more than 100 MFI with more than 1% of samples, ** more than 200 MFI with more than 5% of samples, *** more than 500 MFI with more than 20% of samples and **** more than 1000 MFI with more than 50% of samples.
2. The EBNA-6 peptides contained the whole or part of the EBNA-6 740 peptide (underlined) which was reported to react preferentially with IgG from ME/CFS sera by Loebel et al PLoS ONE 12(6): e0179124 (2017), https://doi.org/10.1371/journal.pone.0179124. The portion marked bold in both EBNA-6 peptides contained the peptide originally reported to be highly antigenic by Falk et al J Med Virol 46, 349-357 (1995), https://www.ncbi.nlm.nih.gov/pubmed/7595412 . 3. The arrow (<-) indicates peptides which were used in several cohort screenings reported in this paper.

Validation of SMIA

The HHV-1, HHV-3, HHV-5 and HHV-6 SMIA whole virus components were validated earlier (Westman et al Front Neurol 2017, doi10.3389/fneur.2017.00040, and Wang et al BMC Infect Dis 2016, doi10.1186/s12879-015-1194-3). Table S2 gives basic evaluation data for HHV-2 whole virus, and HHV-4 VCA p18 peptide 119-148 and EBNA-1 peptide 391-426.

Table S2. Validation of SMIA versus commercial tests

HHV2 (HSV-2)	Whole virus	Focus diagnostics HerpeSelect	100	33, 60, 2, 5	33%
HHV4 (EBV)	Whole virus	Siemens EBNA	13	10, 2, 0, 1	100%
HHV4 (EBV)	Peptide p18 119-148	Siemens EBV VCA	13	9, 1, 0, 3 (borderline Siemens counted as pos)	92%
HHV4 (EBV)	Peptide EBNA1 391-426	Siemens EBV EBNA	13	6, 0, 4, 3	69%
